# C9orf72-Associated Dipeptide Repeat Expansions Perturb ER-Golgi Vesicular Trafficking, Inducing Golgi Fragmentation and ER Stress, in ALS/FTD

**DOI:** 10.1007/s12035-024-04187-4

**Published:** 2024-05-09

**Authors:** Jessica Sultana, Audrey M. G. Ragagnin, Sonam Parakh, Sayanthooran Saravanabavan, Kai Ying Soo, Marta Vidal, Cyril Jones Jagaraj, Kunjie Ding, Sharlynn Wu, Sina Shadfar, Emily K. Don, Anand Deva, Garth Nicholson, Dominic B. Rowe, Ian Blair, Shu Yang, Julie D. Atkin

**Affiliations:** 1 Motor Neuron Disease Research Centre, Faculty of Medicine, Health and Human Sciences, Macquarie University, Sydney, NSW 2109 Australia; 2 Department of Plastic and Reconstructive Surgery, and The Integrated Specialist Healthcare Education and Research Foundation, Macquarie University, Sydney, Australia; 3 ANZAC Research Institute, Concord Hospital, University of Sydney, Sydney, NSW Australia; 4 La Trobe Institute for Molecular Science, La Trobe University, Bundoora, Melbourne, VIC 3086 Australia

**Keywords:** ALS, C9orf72, ER-Golgi transport, ER stress, Omegasome

## Abstract

Hexanucleotide repeat expansions (HREs) in the chromosome 9 open reading frame 72 (*C9orf72*) gene are the most frequent genetic cause of amyotrophic lateral sclerosis (ALS) and frontotemporal dementia (FTD). Both are debilitating neurodegenerative conditions affecting either motor neurons (ALS) in the brain and spinal cord or neurons in the frontal and/or temporal cortical lobes (FTD). HREs undergo repeat-associated non-ATG (RAN) translation on both sense and anti-sense strands, generating five distinct dipeptide repeat proteins (DPRs), poly-GA, -GR, -GP, -PA and -PR. Perturbed proteostasis is well-recognised in ALS pathogenesis, including processes affecting the endoplasmic reticulum (ER) and Golgi compartments. However, these mechanisms have not been well characterised for C9orf72-mediated ALS/FTD. In this study we demonstrate that C9orf72 DPRs polyGA, polyGR and polyGP (× 40 repeats) disrupt secretory protein transport from the ER to the Golgi apparatus in neuronal cells. Consistent with this finding, these DPRs also induce fragmentation of the Golgi apparatus, activate ER stress, and inhibit the formation of the omegasome, the precursor of the autophagosome that originates from ER membranes. We also demonstrate Golgi fragmentation in cells undergoing RAN translation that express polyGP. Furthermore, dysregulated ER-Golgi transport was confirmed in C9orf72 patient dermal fibroblasts. Evidence of aberrant ER-derived vesicles in spinal cord motor neurons from C9orf72 ALS patients compared to controls was also obtained. These data thus confirm that ER proteostasis and ER-Golgi transport is perturbed in C9orf72-ALS in the absence of protein over-expression. Hence this study identifies novel molecular mechanisms associated with the ER and Golgi compartments induced by the C9orf72 HRE.

**Supplementary Information:**

The online version contains supplementary material available at 10.1007/s12035-024-04187-4.

## Introduction

Amyotrophic lateral sclerosis (ALS) is a fatal neurodegenerative disease caused by the loss of motor neurons in the brain, brainstem and spinal cord, leading to muscular weakness and paralysis, and death usually occurs 3–5 years from initial diagnosis [1–3]. ALS shares significant genetic and pathological overlap with frontotemporal dementia (FTD), and the two conditions are thought to represent opposite ends of a disease continuum [4]. FTD affects the frontal and temporal lobes of the cortex, and is characterised by impairment in behaviour, cognitive function, and/or language [5]. A hexanucleotide (GGGGCC) repeat expansion (HRE) in the first intron of the *C9orf72* (*Chromosome 9 open reading frame 72*) gene is the most common genetic cause of both ALS and FTD, although its frequency varies according to geoancestry and is rare in Asian populations [6, 7]. The vast majority of healthy individuals possess ≤ 11 repeats, whereas between thirty and thousands of repeats are found in patients with C9orf72 ALS/FTD [8]. However, many previous studies have detected pathogenic features associated with 24–30 repeats [9].

Repeat associated non-AUG (RAN) translation of the *C9orf72* hexanucleotide repeat expansion results in expression of five different dipeptide repeat proteins (DPRs) that aggregate and form inclusions in affected tissues (DPR: polyGA [glycine-alanine], polyGP [glycine-proline], polyGR [glycine-arginine], polyPA [proline-alanine] and polyPR [proline-arginine]) [10, 11]. DPR inclusions are most prominent in the cerebellum, hippocampus, and neocortex, and whilst each DPR displays a similar anatomical distribution [12–14], polyGA and polyGP are the most abundant DPRs in post-mortem tissues (poly-GA > GP > GR > PR/PA) [15]. In addition, polyGP has been detected in the CSF of asymptomatic individuals carrying C9orf72 mutations, before significant degeneration occurs [16]. Similarly, more recently, polyGR and polyGA were also detected in the CSF of ALS patients [17]. The HRE is thought to induce toxicity by both loss and gain of function mechanisms. Gain of toxic function is mediated by both expression of the DPRs and production of the greatly expanded RNAs that accumulate into RNA foci [18]. Loss of function resulting from C9orf72 haploinsufficiency leads to reduced expression of C9orf72 protein in ALS/FTD patients, which is also implicated in neurodegeneration [19]. Many studies have linked DPRs to toxicity in neurons [10, 11, 20–22].

Several pathogenic mechanisms have been described in C9orf72-ALS/FTD, including nuclear dysfunction, altered RNA splicing and RNA granule dynamics, impaired nucleocytoplasmic transport, mitochondrial dysfunction [23], DNA damage [24] and disruption of the proteostasis network [25, 26]. The endoplasmic reticulum (ER) is an important part of the proteostasis network, and the first compartment of the secretory pathway where secretory, transmembrane, and extracellular proteins are folded before being transported to the Golgi apparatus. ALS-linked mutations in several proteins (SOD1, TDP-43, FUS, UBQLN2) inhibit transport between the ER and the Golgi apparatus [27, 28]. However, this mechanism has not yet been described for C9orf72-ALS/FTD.

ER-Golgi transport is initiated when vesicles containing protein cargo bud from the ER coated in coat protein II (COPII) complex, consisting of Sec31, Sec13, Sec23, Sec24 and Sar1. After the budding, fusion to target membranes at the Golgi follows [29, 30]. Efficient ER-Golgi transport is required to maintain ER homeostasis, and dysfunction to the ER induces ER stress. This results in activation of the unfolded protein response (UPR), which aims to restore ER proteostasis. However, prolonged activation of the UPR initiates apoptotic cell death to protect the organism from the load of misfolded proteins via up-regulation of C/EBP homologous protein (CHOP) [31–33]. Perturbations in ER-Golgi transport can also induce fragmentation of the Golgi apparatus [34]. Correct functioning of the Golgi apparatus is closely linked to cellular viability [35] and its unique morphology, with tethered stacks of cisternae, is required for proper function. However, the Golgi apparatus can fragment into condensed, tubulovesicular punctate structures when its functions are compromised or during pathological conditions [26, 35, 36].

C9orf72 is a member of the Differentially Expressed in Normal and Neoplasia (DENN) family of proteins [37–39], that function in vesicular pathways as either guanosine exchange factors (GEFs) or GTPase activating proteins (GAPs) for small GTPases, including Rab proteins [40, 41]. The normal cellular function of C9orf72 involves Rab-mediated endosomal trafficking and initiation of autophagy [42, 43], although recently, C9orf72 was also shown to regulate mitochondrial oxidative phosphorylation and hence energy homeostasis [44]. Autophagy is another important component of proteostasis that involves degradation of proteins within the lysosome. Macro-autophagy is a major type of autophagy involving the formation of double-membrane vesicles, or ‘autophagosomes’. The ER/UPR is functionally linked to macro-autophagy, because a subdomain of the ER membrane produces the ‘omegasome’, a membrane platform from which phagophores, the precursors of mature autophagosomes, are formed [45–48]. Autophagy is also activated by induction of ER stress [49–51]. During autophagy initiation, C9orf72 interacts with the Unc-51-like kinase 1 (ULK1) protein kinase complex, acting as a Rab1 effector [43], which in turn associates with omegasomes [52]. Autophagy counteracts accumulation of the C9orf72 DPRs in ALS, whereas impaired autophagy drives the accumulation of DPRs [53]. The C9orf72 HRE can perturb autophagy, but this has been disputed [54, 55].

In this study we examined whether the C9orf72 DPRs perturb the ER and Golgi compartments in neuronal cells. We demonstrate that DPRs poly-GA, -GR, -GP inhibit ER-Golgi transport and the formation of the omegasome, and they induce Golgi fragmentation, ER stress, and apoptosis. These findings therefore provide novel insights into disease mechanisms induced by the C9orf72 HREs in ALS/FTD.

## Methods

### Constructs

Codon optimized cDNA constructs encoding the C9orf72 DPRs (polyGA_x40_, polyGR_x40_, polyGP_x40_, polyPR_x40,_ polyPA_x40_), tagged with the FLAG epitope at the amino terminus, were subcloned into pcDNA3 (Genscript) using BamH1 and EcoRV. Expression of each DPR was driven by the presence of an initiation codon, ATG. A second set of constructs were designed containing either G4C2_x0_, G4C2_x3_ and G4C2_x40_ repeats, tagged with mCherry, FLAG, HA and MYC epitopes at the amino terminus, and were also subcloned using BamH1 and EcoRV into pcDNA3 (Genscript). These constructs, G4C2_RANx0,_ G4C2_RANx3, or_ G4C2_RANx40,_ were created without an ATG codon, so that they can perform RAN translation in any forward reading frame. The *XBP-1-Venus* construct was a kind gift from Dr Masayuki Miura [56]. The constructs encoding mutant vesicular stomatitis viral glycoprotein (VSVG^ts045^), fused to either mCherry in pEGFPdKA206K-N1-mCherry vector or VSVG^ts045^ fused to EGFP in pEGFP-C1, were a kind gift from Dr Jennifer Lippincott-Schwartz (National Institutes of Health, Bethesda, USA). The EGFP-Double FYVE-containing protein 1 (DFCP1) in pEGFP-C2 (Invitrogen) was a kind gift from Dr Nicholas Ktistakis, Babraham Institute, Cambridge, England, UK [52].

### Cell Culture and Transfection

Mouse neuroblastoma Neuro2A cells (obtained from the American Type Culture Collection) were cultured to 80% confluence in Dulbecco’s modified Eagle’s medium (DMEM) with 10% (v/v) foetal calf serum (FCS) at 37 °C in a humidified atmosphere of 5% CO_2_. Cells were then transfected with plasmids encoding either FLAG-tagged C9orf72 DPRs (polyGA_x40_, polyGR_x40_, polyGP_x40_, polyPR_x40_) or G4C2_RANx0,_ G4C2_RANx3, or_ G4C2_RANx40,_ using Lipofectamine™ 2000 (Invitrogen) according to the manufacturer’s instructions. Cells were also co-transfected with these constructs (or pcDNA3.1 empty vector [EV]) and either VSVG^ts045^ or Venus-tagged XBP-1 (or pcDNA3.1 EV) using Lipofectamine 2000™ (Invitrogen) according to the manufacturer’s instructions.

### Human Fibroblasts

Human dermal fibroblasts from one control and one familial ALS (FALS) C9orf72 patient carrying the hexanucleotide GGGGCC repeat expansion were examined. The FALS patient (male, 66 years old) developed weakness and wasting first in the distal lower limbs, then subequently in the upper limbs, which was associated with gross fasciculations. Electromyography and nerve conduction studies were consistent with an axonal neuropathy and anterior horn disease. Progression was rapid with a disease duration of 9 months. A neurologically normal individual was used as a control. To examine Sec 31 positive clusters, the same patients and two neurologically normal controls (81 years old male and 65 years old female) were used in immunocytochemistry experiments. Fibroblasts were maintained in T-75 flasks in DMEM supplemented with 10% (v/v) foetal bovine serum (FBS) and 1% Penicillin–Streptomycin following the manufacturer’s protocol. Cells were incubated at 37 °C under 5% CO2 and passaged before reaching confluence.

### Immunocytochemistry

Neuro2A cells grown on 13 mm coverslips were washed in 0.1 M phosphate-buffered saline (PBS), pH 7.2 and fixed in 4% paraformaldehyde (PFA) in PBS for 10 min. After two washes in PBS, cells were permeabilised in 0.1% (v/v) Triton X-100 in PBS for 10 min and incubated in blocking buffer (3% [wt/v] bovine serum albumin [BSA, Sigma] in PBS) for 30 min with gentle rocking. Cells were then incubated overnight at 4 °C in primary antibodies diluted in 1% (wt/v) BSA in PBS: monoclonal mouse anti-FLAG (1:250, Sigma F1804), anti-GADD153/CHOP (1:50; Santa CruzSc-7351), anti-GM130 (1:50, BD Transduction Laboratories 610823), anti-calnexin (1:200, Abcam ab22595), anti-KDEL (1:400, Abcam ab12223), anti-Sec31A (1:100, Sigma HPA005457; 1:200 BD Transduction Laboratories 612351), or polyclonal rabbit anti-C9orf72 DRP antibodies; GA repeat (1:400, Proteintech 24492–1-AP), GR repeat (1:400, Proteintech 23978–1-AP), GP repeat (1:400, Proteintech 24494–1-AP), or PR repeat (1:400, Proteintech 23979–1-AP). After 3 5-min washes in PBS, cells were incubated for 1 h in appropriate secondary antibodies in PBS coupled to either Alexa 488, Alexa 594, or Alexa 647 (1:200, Invitrogen). Cells were then washed as above and stained with Hoechst 33342 (1:10,000; Invitrogen) in distilled H2O and mounted on slides in fluorescent mounting medium (Dako). Representative images were acquired with a Zeiss 780 confocal laser scanning microscope (Zeiss) at 100x/na = 1.46 magnification, while low quality images for cell counts were acquired at 100x/na = 1.6 and 40x/na = 1.3 magnifications for quantitation with an epi-fluorescence microscope (Zeiss) equipped with a digital CCD camera (Zeiss). Images for analysis of Sec31A positive clusters in primary fibroblasts were acquired with a Leica Stellaris 5 confocal laser microscope (Leica Biosystems) equipped with a supercontinuum white light laser (440–790 nm). Spectral positions were optimized and sequentially acquired in separate tracks to prevent bleed through effects. Images were acquired at 63 × objective magnification with a pixel area of 0.002 µm^2^ and multiple Z-stacks were obtained at 0.3 µm intervals to cover the depth of cells. Maximum intensity projections of Z-stacks were converted to image files using the Leica Application Suite (LAS) X Office version 1.4.4.26810 (Leica Biosystems).

### Immunohistochemistry

Five micrometer paraffin-embedded, formalin-fixed human post-mortem cervical spinal cord tissue sections from three neurologically normal controls and three ALS patients with C9orf72 repeat expansions (obtained from the NSW Brain Tissue Resource Centre, collected at autopsy) were used to examine Sec31A morphology. Patient demographic information is detailed in Table 1. Fluorescent immunohistochemistry was performed as previously described [28]. Briefly, tissue sections were dewaxed and rehydrated in xylene and a graded ethanol series of decreasing concentrations. Antigen retrieval was performed by boiling tissues in citric acid buffer at 100 °C for 30 min. Tissues were then immunolabelled with primary anti-Sec31A antibody (1:100, Sigma HPA005457), followed by AlexaFluor 555 conjugated secondary antibody (1:250, Invitrogen). Nuclei were counterstained with DAPI (1:5,000, Invitrogen) and tissues were mounted with fluorescent mounting medium (Dako). At least five neurons per case were imaged using a Zeiss LSM 880 inverted confocal laser scanning microscope (Zeiss) at 100 × /na = 1.46 magnification. Neurons in Sec31A-channel images were selected using the freehand selection tool on Fiji Image J, excluding lipofuscin. This work was approved by the Macquarie University Human Ethics Committee (Ref: 5201600719).

**Table 1:** Demographical information of control and ALS patients used in the immunohistochemical study

Classification	Gender	Age	Disease duration (yr)	Age of onset	Post-mortem interval (hr)
Control 1	Male	61	N/A	N/A	30
Control 2	Female	62	N/A	N/A	35
Control 3	Male	75	N/A	N/A	34
ALS-C9orf72 1	Female	64	2	62	7
ALS-C9orf72 2	Male	75	1	74	21.5
ALS-C9orf72 3	Male	60	2	58	99

### Western Blotting

Neuro2A cells were lysed in 50 mM Tris–HCl (pH 7.6), 150 mM NaCl, 0.1% [v/v] NP40, 0.1% [wt/v] sodium dodecyl sulphate (SDS) and 1% protease inhibitor cocktail (Sigma) and incubated on ice for 10 min. Cells were then centrifuged at 16,000 g for 10 min and the supernatant collected. Protein lysates were loaded (40 μg total protein) into 10% SDS–polyacrylamide gels and transferred onto nitrocellulose membranes. After 30 min in 5% (wt/v) skim milk in TBS, the membrane was incubated overnight at 4 °C with primary antibodies in 5% (wt/v) skim milk in TBS and 0.1% Tween20 (TBST): monoclonal mouse anti-GAPDH (1:1000, Ambion AM4300), anti-C9ORF72 (1:100, SantaCruz, sc-138763), or polyclonal rabbit anti-C9orf72 DPR GP (1:1000, Proteintech 24494–1-AP) antibodies in 5% (w/v) skim milk in TBST. After 3 washes in TBST, membranes were incubated at room temperature for 1 h in the appropriate secondary antibodies; HRP-conjugated goat anti-mouse (1:2,000, Chemicon), HRP-conjugated goat anti-rabbit (1:2,000, Millipore, AP132P); in 5% (w/v) skim milk in TBST and developed using advanced chemiluminescent reagents (Roche) according to the manufacturer’s instructions.

### VSVG^ts045^ Transport Assay

Neuro2A cells and control or C9orf72 patient fibroblasts were co-transfected with mCherry- or GFP-tagged VSVG^ts045^ for 72 h. The medium was changed 4 h after transfection and cells were incubated overnight at 40 °C (restrictive temperature) to reversibly misfold VSVG^ts045^, which leads to its accumulation within the ER. Cells were then incubated in DMEM containing cyclohexamide (Sigma, 01810, 20 μg/mL) to block protein synthesis at 32 °C (permissive temperature) for 30 min. Cells were then washed in PBS and fixed in 4% PFA and processed for immunocytochemistry using anti-KDEL, anti-calnexin (CNX) or anti-GM130 antibodies as described above. Images were acquired using a 100x/na = 1.6 objective lens on an epi-fluorescence microscope (Olympus), equipped with a monochrome camera.

### Quantitative Analysis of the Co-Localisation of VSVG^ts045^ with Calnexin/KDEL or GM130 in Neuronal Cells

Mander’s coefficient was used to calculate the degree of colocalisation between the fluorescent pixels of mCherry- or GFP-tagged VSVG^ts045^ and the immunofluorescent pixels of calnexin/KDEL or GM130. Sixty transfected Neuro2A cells per group from three independent experiments, or from n = 38 fibroblasts from a healthy control and n = 35 fibroblasts from an ALS patient bearing a mutation in *C9orf72,* were examined using the ImageJ JaCoP plugin [57]. The resulting Mander’s correlation coefficient ranged from 0: no co-localisation to 1: high co-localisation [58].

### Quantitative Analysis of the Average Sec31A Puncta

For post-mortem tissue, images were converted to 8-bit to generate a binary image and the threshold level was then set to 53–255, followed by the ‘Analyse particles’ function on Fiji Image J. The ‘average particle size’, ‘Count’ and ‘% Area’ was extracted from the result summary and used as an indication of Sec 31 puncta morphology. The data was analysed to control for heterogeneity of human samples using the random effects model to compare the average Sec31A puncta area between controls and ALS patients in Graph Pad Prism 8.

For primary fibroblasts, the maximum intensity projection of Z-stacks was converted to an image file for analysis on Fiji ImageJ. To combine closely spaced particles as clusters and reduce noise, the ‘Despeckle’ function in ImageJ was used. Following this, the threshold level was set (50–255) and measurements obtained from the ‘Analyze Particles’ function as above. The ‘Area’ was used as an indicator of size, ‘Circularity’ as indicator of shape, and ‘Raw Integrated Density’ as intensity of staining in clusters. Statistics were performed using individual clusters as replicates and a t-test was performed between the C9orf72 and control samples. The findings were technically replicated using two different antibodies to Sec31A (mouse IgG1 targeting human Sec31A aa. 522–719 and rabbit polyclonal antibody targeting recombinant protein epitope signature tag aa. 648–783 of human Sec31A isoform 4).

### Quantitative Analysis of Golgi Fragmentation in Neuronal Cells

Fragmentation of the Golgi apparatus was identified by the detection of punctate, dispersed GM130-positive structures in contrast to the typical condensed perinuclear ribbon-like Golgi in healthy cells. It was scored binary by a blinded single evaluator following immunocytochemistry with anti-GM130 antibody using ImageJ as previously described [27, 28]. The percentage of transfected cells with fragmented Golgi was quantified from at least 100 Neuro2A cells per group from three independent experiments. Only cells where the Golgi structure was clearly visible were analysed.

### Quantitative Analysis of XBP1 and CHOP Nuclear Immunoreactivity in Neuronal Cells

The percentage of cells displaying nuclear immunoreactivity to CHOP or XBP1 was quantified using ImageJ from at least 100 Neuro2A cells per group expressing C9orf72 DPRs from n = 3 independent experiments.

### Quantitative Analysis Of Apoptotic Nuclei in Neuronal Cells

Apoptotic nuclei were defined as condensed (under 5 µm in diameter) or fragmented (multiple condensed Hoechst-positive structures in one cell) as previous [31, 59]. The percentage of apoptotic cells was quantified from at least 100 Neuro2A cells per group expressing C9orf72 DPRs from 3 independent experiments. Cells undergoing cell division were excluded from analysis.

### Statistics

Data are presented as mean value ± standard error of the mean (SEM). Statistical comparisons between group means were performed using GraphPad Prism software (Graph Pad software, Inc.), using Student’s *t*-test or one-way ANOVA, followed by post hoc Tukey test for multiple comparisons when justified. The significance threshold was set at p = 0.05. To examine Sec31A puncta staining in spinal cord motor neurons, a linear mixed effect model, as described previously [60], was fitted to assess whether there was an association between Sec31A staining pattern and whether an individual was classified as a control or a positive ALS patient. Sample identification numbers were included as a random effect in the model to account for multiple observations from the same individual. R V.3.5.1 and the ‘lmerTest’ package were used for this analysis.

## Results

### Expression and Cellular Localisation of C9orf72 Codon-Optimised DPRsx40 in Neuro2A Cells

**Fig. 1:**
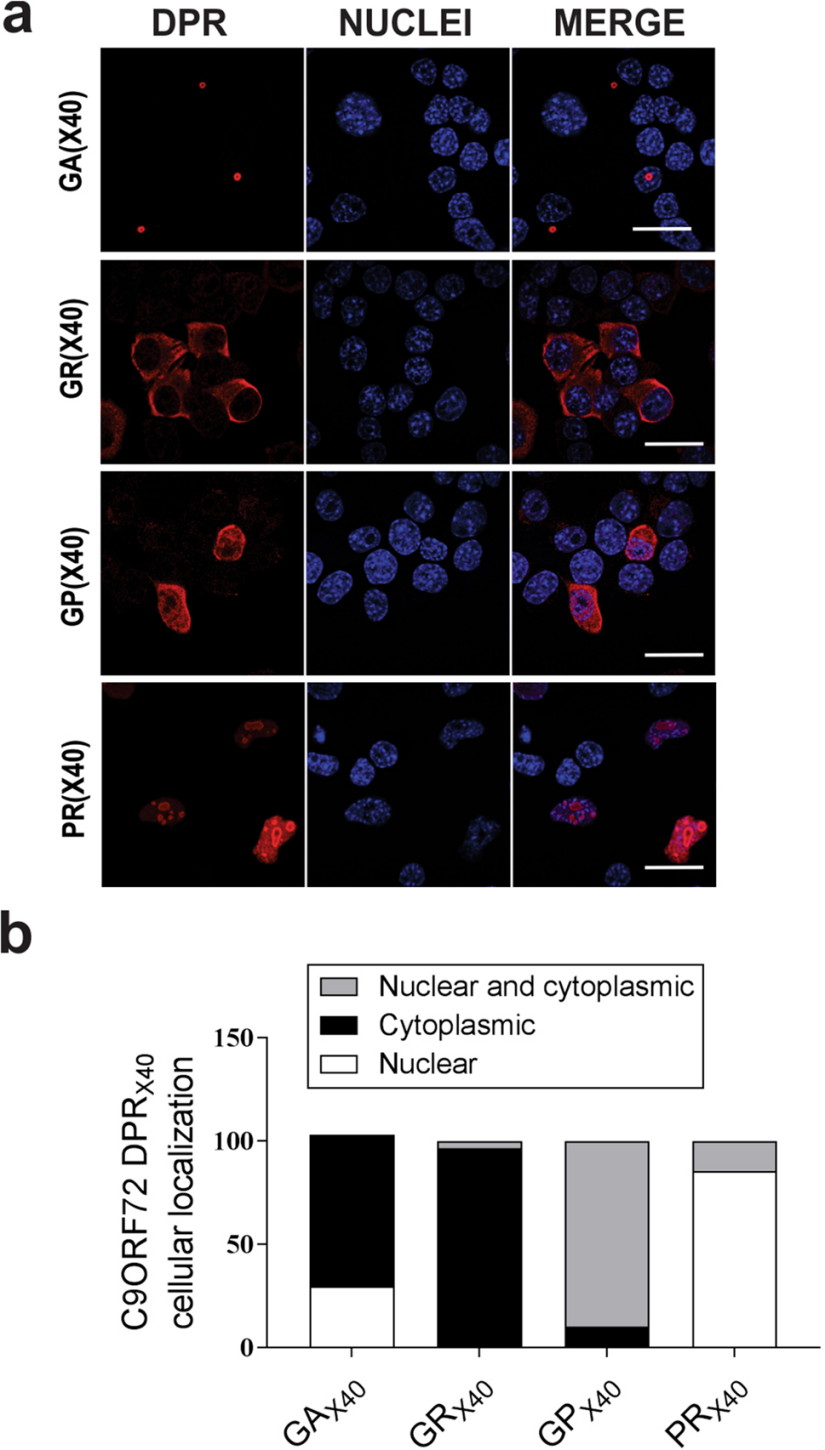
Expression and cellular localisation of C9orf72 codon-optimised DPRs in Neuro2A cells. (**A**) Fluorescent confocal microscopy images following immunocytochemistry for C9orf72 DPRs in untransfected cells (UT), cells expressing pcDNA3.1 empty vector (EV), or codon-optimised C9orf72 DPRs_x40_ for 48 h. Scale bar: 10 µm. Antibodies specific for each DPR were used (polyGA, polyGR, polyGP, or polyPR). (**B**) Quantification of the percentage of cells with nuclear only, cytoplasmic only, and both nuclear and cytoplasmic localisation, C9orf72 DPRs from the images in (A). Mean ± SEM, n = 3, 100 + cells per group were examined

Codon-optimized *C9orf72* DPR constructs containing an ATG initiation codon were designed to express 40 repeats of polyGA, polyGR, polyGP, or polyPR separately. A construct encoding polyPA did not express reliably in initial experiments, hence it was not studied further. The cellular localisation of each C9orf72 DPR was then examined in lysates prepared from Neuro2A cells transfected for 48 h, by immunocytochemistry using specific anti-GA, anti-GR, anti-GP and anti-PR antibodies [61–63]. Confocal microscopy confirmed expression of each C9orf72 DPR (Fig. 1A). Cells were then categorised according to where each C9orf72 DPR was expressed; nucleus only, cytoplasm only or both nucleus and cytoplasm (Fig. 1A, B). PolyGA was detected mainly in the cytoplasm only (73.4 ± 5.6%) although a proportion was expressed in the nucleus only (29.7 ± 8.5%), and similarly, polyGR was expressed predominantly in the cytoplasm (96.6 ± 1%). In contrast, whilst polyGP was expressed in both the nucleus and the cytoplasm (89.9 ± 5.3%), polyPR was predominantly expressed in the nucleus only (85.5 ± 1%). Hence, the four C9orf72 DPRs examined here were found to display different cellular localizations in neuroblastoma cells, consistent with previous studies [22, 64–66].

### C9orf72 DPRs polyGA, polyGR and polyGP Inhibit ER-Golgi Transport in Neuro2A Cells

**Fig. 2:**
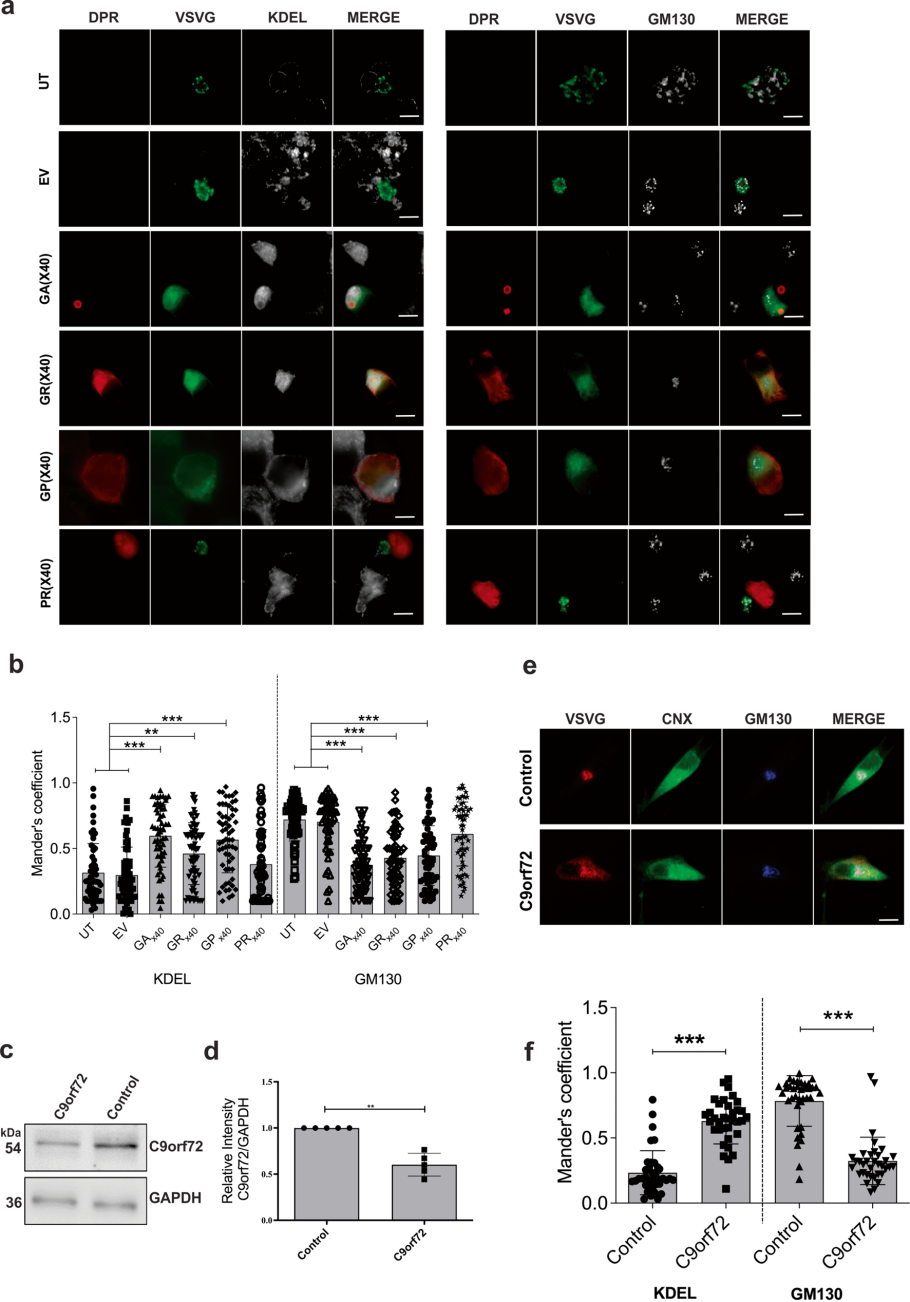
C9orf72 DPRs disrupt ER-Golgi transport in neuronal cells and in C9orf72 patient fibroblasts. (**A**) Fluorescent confocal microscopy images of C9orf72 DPRs and GFP-tagged VSVG^ts045^ following immunocytochemistry for C9orf72 DPRs using polyGA, polyGR, polyGP or polyPR antibodies with KDEL (as an ER marker) or GM130 (as a Golgi marker) antibodies, in untransfected cells (UT) or in cells co-expressing VSVG^ts045^ and pcDNA3.1 empty vector (EV) or codon-optimised C9orf72 DPRs_x40_, 30 min after incubation for 12 h at 40 °C and 30 min at the permissive temperature (32 °C). Scale bar: 10 µm. (**B**) Quantification of the degree of co-localisation of VSVG^ts045^ with either KDEL or GM130 of images in (A) using Mander’s coefficient (0: no colocalisation and 1: high colocalisation). Mean ± SEM, one-way ANOVA followed by a *post-hoc* Tukey test. n ≥ 60 transfected Neuro2A cells per group from 3 independent experiments were examined: **p < 0.01, ***p < 0.001. VSVG^ts045^ is retained in the ER and less is transported to the Golgi apparatus in cells expressing C9orf72 DPRs. (**C**) Western blotting using an anti-C9orf72 antibody in lysates prepared from C9orf72 patient fibroblasts. GAPDH was used as a loading control. **(D)** Quantification of blots in (C). C9orf72 protein levels were significantly lower in ALS patient fibroblasts compared to controls, **p < 0.01, n = 5, Mean ± SEM, students t test. (**E**) Fluorescence microscopy images of mCherry-tagged VSVG^ts045^ and the ER and the Golgi apparatus following immunocytochemistry for calnexin (CNX) and GM130 respectively in fibroblasts from control and C9orf72 patients. Scale bar: 10 µm. (**F**) Quantification of the degree of co-localisation of mCherry-tagged VSVG^ts045^with either the ER (calnexin) or the Golgi apparatus (GM130) of images in (D) using Mander’s coefficient. Mean ± SEM, t-test, n = 38 fibroblasts from 2 controls, n = 35 fibroblasts from 2 patients bearing a mutation in *C9orf72*; ***p < 0.001. VSVG^ts045^ is retained in the ER and less is transported to the Golgi apparatus in C9orf72 patient fibroblasts

We next examined whether protein trafficking between the ER and Golgi compartments is inhibited in cells expressing C9orf72 DPRs. For this study, the temperature sensitive mutant vesicular stomatitis glycoprotein-ts045 (VSVG^ts045^), a widely employed classical marker to monitor ER–Golgi trafficking, was used [67]. At 40 °C VSVG^ts045^ misfolds and is retained within the ER. Once the temperature is shifted to 32 °C (permissive temperature) VSVG^ts045^ refolds and traffics to the Golgi apparatus. Immunocytochemistry of cells co-expressing C9orf72 DPRs with GFP-tagged VSVG^ts045^ was then performed using antibodies specific for each DPR and markers of the ER (KDEL) or Golgi (GM130) to examine the localisation of VSVG^ts045^ in each compartment (Fig. 2A). Subsequent analysis using Mander’s coefficient (where 0 indicated no co-localisation and 1: high co-localisation) revealed a low degree of co-localisation between mCherry-VSVG^ts045^ and KDEL in untransfected cells (UT) (0.32 ± 0.03) and in cells expressing empty vector (EV) only (0.30 ± 0.03), demonstrating that little VSVG^ts045^ was retained in the ER in these populations (Fig. 2B). In contrast, significantly more co-localisation was observed between VSVG^ts045^ and KDEL in cells expressing polyGA (0.60 ± 0.03, *p* < 0.001), polyGR (0.46 ± 0.03, *p* < 0.01) and polyGP (0.57 ± 0.03, *p* < 0.001), compared to UT and EV cells, demonstrating that more VSVG^ts045^ was retained in the ER (Fig. 2B). However, there was no significant difference in cells expressing polyPR to controls (*p* > 0.05). Similar analysis of Golgi co-localisation using Mander’s coefficient revealed a high degree of co-localisation between VSVG^ts045^ and GM130 in UT cells (0.72 ± 0.02) and cells expressing EV (0.70 ± 0.03), demonstrating that VSVG^ts045^ was efficiently transported to the Golgi in these populations. In contrast, significantly less co-localisation was observed between VSVG^ts045^ and GM130 in cells expressing C9orf72 polyGA (0.38 ± 0.02, *p* < 0.001), polyGR (0.43 ± 0.03, *p* < 0.001) or polyGP (0.45 ± 0.03, *p* < 0.001), compared to UT and cells expressing EV (Fig. 2B). However, the degree of co-localisation between VSVG^ts045^ and GM130 was not significantly different between cells expressing polyPR ( 0.14, *p* > 0.05) compared to EV and UT cells. These findings indicate that VSVG^ts045^ is retained in the ER and not properly transported to the Golgi apparatus in cells expressing polyGA, polyGR or polyGP, representing the DPRs with the most cytoplasmic localisation. Hence these ALS/FTD-associated C9orf72 DPRs inhibit ER-Golgi transport in neuronal cells. However, polyPR, which displayed more nuclear localisation than the other DPRs, did not inhibit ER-Golgi transport.

To validate these results in a more physiological setting and in the absence of protein over-expression, we then examined ER-Golgi trafficking in fibroblasts cultured from an ALS patient bearing the C9orf72 repeat expansion and a control patient. The C9orf72 fibroblasts expressed significantly less (0.6-fold, p < 0.01) C9orf72 compared to control fibroblasts (Fig. 2C, D), consistent with previous studies [68]. Fibroblasts were transfected with mCherry-tagged VSVG^ts045^ and immunocytochemistry using antibodies against either calnexin as an ER marker and GM130 as a Golgi marker was performed. Mander’s coefficient revealed significantly (*p* < 0.001) more co-localisation between VSVG^ts045^ and calnexin in C9orf72 patient fibroblasts (0.63 ± 0.03) compared to control fibroblasts (0.23 ± 0.03) (Fig. 2E, F), demonstrating that VSVG^ts045^ was retained in the ER in these cells. Conversely, significantly (*p* < 0.001) less co-localisation between VSVG^ts045^ and GM130 was detected in C9orf72 patient fibroblasts (0.32 ± 0.03) compared to controls (0.78 ± 0.03), demonstrating that transport of VSVG^ts045^ to the Golgi was impaired (Fig. 2E, F). These data therefore provide further evidence that ER-Golgi transport is impaired in C9orf72-ALS.

### C9orf72 DPRs GA, GR and GP Induce ER Stress and Apoptosis in Neuro2A Cells

**Fig. 3:**
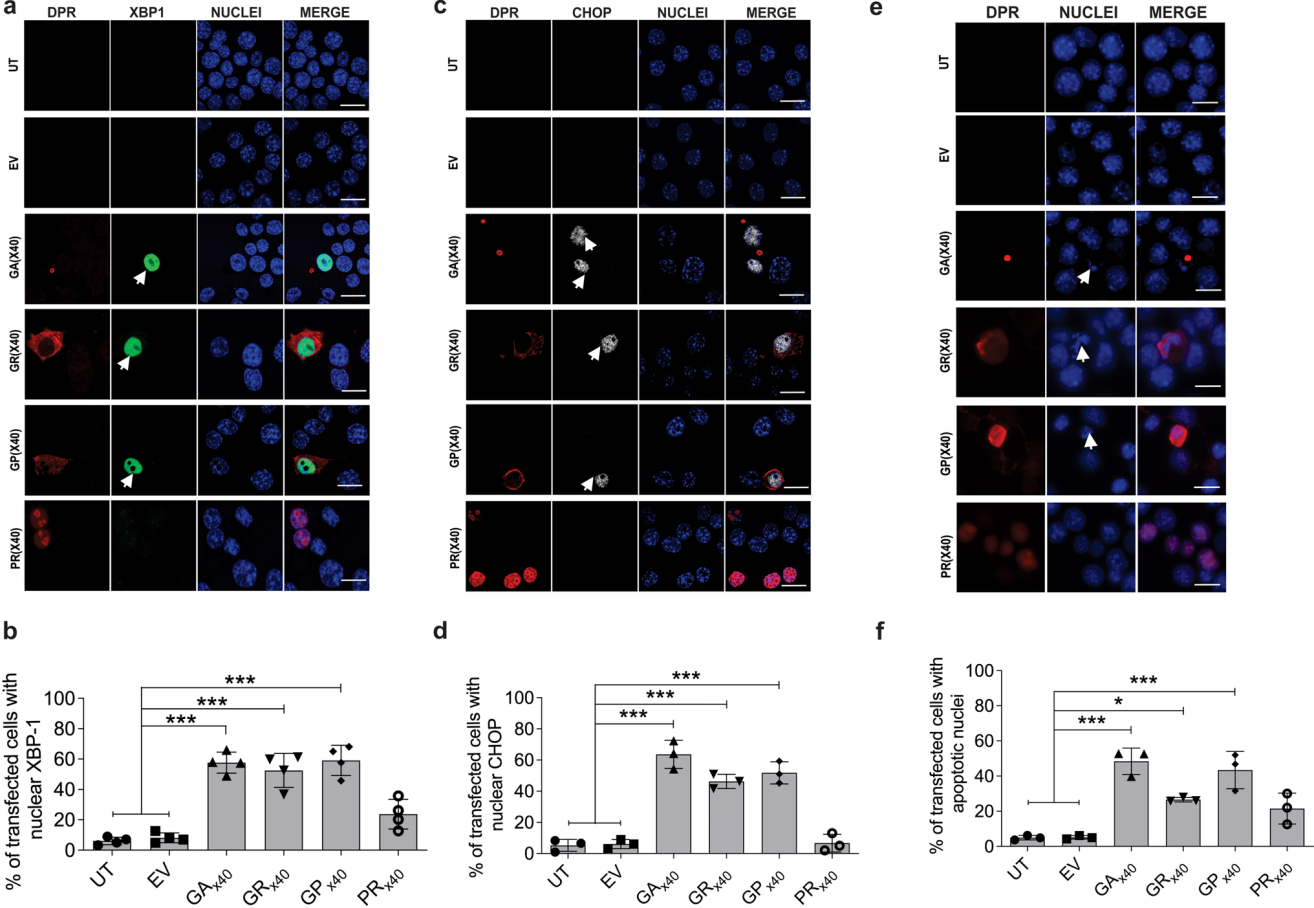
C9orf72 DPRs GA, GR and GP induce ER stress and apoptosis in Neuro2A cells. (**A**) Fluorescent confocal microscopy images of Venus-tagged XBP1 and codon-optimised C9orf72 GA_x40_, GR_x40_, GP_x40_ or PR_x40_, following immunocytochemistry for C9orf72 DPRs, and Hoechst staining of Neuro2A cells expressing EV or C9orf72 DPR_x40_. Scale bar: 10 µm. Activation of the UPR is indicated by XBP1 expression in the nucleus (white arrows). (**B**) Quantification of the proportion of transfected cells with nuclear XBP1 expression in (**A**). Mean ± SEM, one-way ANOVA followed by *post-hoc* Tukey’s test, n = 4; ***p < 0.001. Significantly more cells with nuclear XBP1 immunoreactivity are present in populations expressing 40 copies of C9orf72 polyGA, polyGR or polyGP DPRs compared to UT and EV cells. (**C**) Fluorescent confocal microscopy images of codon-optimised C9orf72 GA_x40_, GR_x40_, GP_x40_ or PR_x40_ following immunocytochemistry for C9orf72 DPRs and CHOP, and Hoechst staining in cells expressing EV or codon-optimised C9orf72 GA_x40_, GR_x40_, GP_x40_ or PR_x40_. Scale bar: 10 µm. Activation of the UPR is indicated by CHOP immunoreactivity in the nucleus (white arrows). (**D**) Quantification of the proportion of transfected cells with nuclear CHOP. Mean ± SEM, one-way ANOVA followed by *post-hoc* Tukey test, n = 3; ***p < 0.001. Significantly more cells with nuclear CHOP immunoreactivity are present in populations expressing C9orf72 polyGA_40_, polyGR_40_ or polyGP_x40_ DPRs compared to UT and cells expressing EV only. (**E**) Fluorescent microscopy images of codon-optimised FLAG-tagged C9orf72 polyGA, polyGR, polyGP or polyPR DPRs_x40_ following immunocytochemistry for C9orf72 DPRs. Arrows: condensed nucleus. Scale bar: 10 µm. (**F**) Quantification of the proportion of transfected cells in (**E**) with nuclear condensation, indicating apoptosis is underway. Mean ± SEM, n = 3; one-way ANOVA followed by *post-hoc* Tukey test, *p < 0.05, ***p < 0.001

We next examined whether impairment of ER-Golgi transport was coincident with ER stress. For this purpose we used two key markers of UPR activation: XBP1 and CHOP, as previously described [27, 67]. During ER stress, XBP1 is spliced to form an active transcription factor that moves into the nucleus, where it regulates genes involved in the UPR [69]. A specific reporter construct was used, *XBP1-Venus,* whereby Venus expression is only detected if XBP-1 is spliced [56]. Immunocytochemistry using anti-DPRs antibodies of Neuro2A cells co-expressing C9orf72 DPRs and XBP1-Venus for 48 h was performed with counterstaining for Hoechst 33342 (Fig. 3A). Quantification revealed that few UT cells (6.1 ± 1.2%) and cells expressing EV (8.1 ± 1.6%) displayed nuclear XBP1-Venus (Fig. 3B). Significantly (*p* < 0.001) more cells with nuclear XBP1 were detected in populations expressing C9orf72 DPRs polyGA (57.7 ± 3.5%), polyGR (52.5 ± 5.6%) and polyGP (59.1 ± 5.0%), compared to UT and EV (Fig. 3B). However, no significant differences were detected in cells expressing polyPR (23.7 ± 4.9%) compared to UT and EV controls (*p* > 0.05). Hence these data reveal that polyGA, polyGR and polyGP induce ER stress, unlike polyPR.

CHOP is a transcription factor that displays nuclear immunoreactivity and can be used to monitor the apoptotic phase of the UPR [27, 28, 70, 71]. Immunocytochemistry of Neuro2A cells expressing C9orf72 DPRs for 48 h was performed using anti-DPRs and anti-CHOP antibodies (Fig. 3C). Quantification revealed that whilst few cells with nuclear CHOP were present in UT (5.3 ± 2.2%) and EV populations (6.1 ± 1.7%), significantly (*p* < 0.001) more were detected in those groups expressing C9orf72 polyGA (63.6 ± 5.2%), polyGR (46.3 ± 2.6%) or poly GP (51.8 ± 4.1%) (Fig. 3D). However, no significant differences were detected in cells expressing polyPR (6.7 ± 3.3%) compared to UT and EV controls (*p* > 0.05). These data therefore confirm that C9orf72 DPRs with cytoplasmic localisation, polyGA, polyGR and polyGP, but not polyPR with more nuclear localisation, induce ER stress. These findings therefore correlate with impairment of ER-Golgi transport by the same DPRs in neuronal cells.

Given that CHOP is pro-apoptotic [72, 73], we next examined whether activation of CHOP correlates with activation of apoptosis in cells expressing the C9orf72 DPRs. Apoptosis was analysed by examining the presence of apoptotic, condensed nuclei, as previously described [31, 59, 74, 75]. Immunocytochemistry using anti-DPR antibodies and counter-staining with Hoechst (to identify the nuclei) was performed (Fig. 3E). Quantification of the percentage of transfected cells with condensed, apoptotic nuclei revealed that few apoptotic cells were present in UT (4.9 ± 0.8%) and EV populations (5.2 ± 0.6%) (Fig. 3F). In contrast, significantly more apoptotic cells were present in populations expressing C9orf72 DPRs polyGA (48.4 ± 4.3%, *p* < 0.001), polyGR (26.6 ± 0.7%, *p* < 0.05) and polyGP (43.4 ± 6.1%, *p* < 0.001) compared to UT and cells expressing EV. However, no significant differences were detected in cells expressing polyPR (21.5 ± 5.1%) compared to UT and EV controls (*p* > 0.05). These data therefore confirm that C9orf72 DPRs polyGA_,_ polyGR and polyGP, but not polyPR, induce CHOP and apoptosis in Neuro2a cells.

### C9orf72 DPRs polyGA and polyGP Disrupt Omegasome Formation

**Fig. 4:**
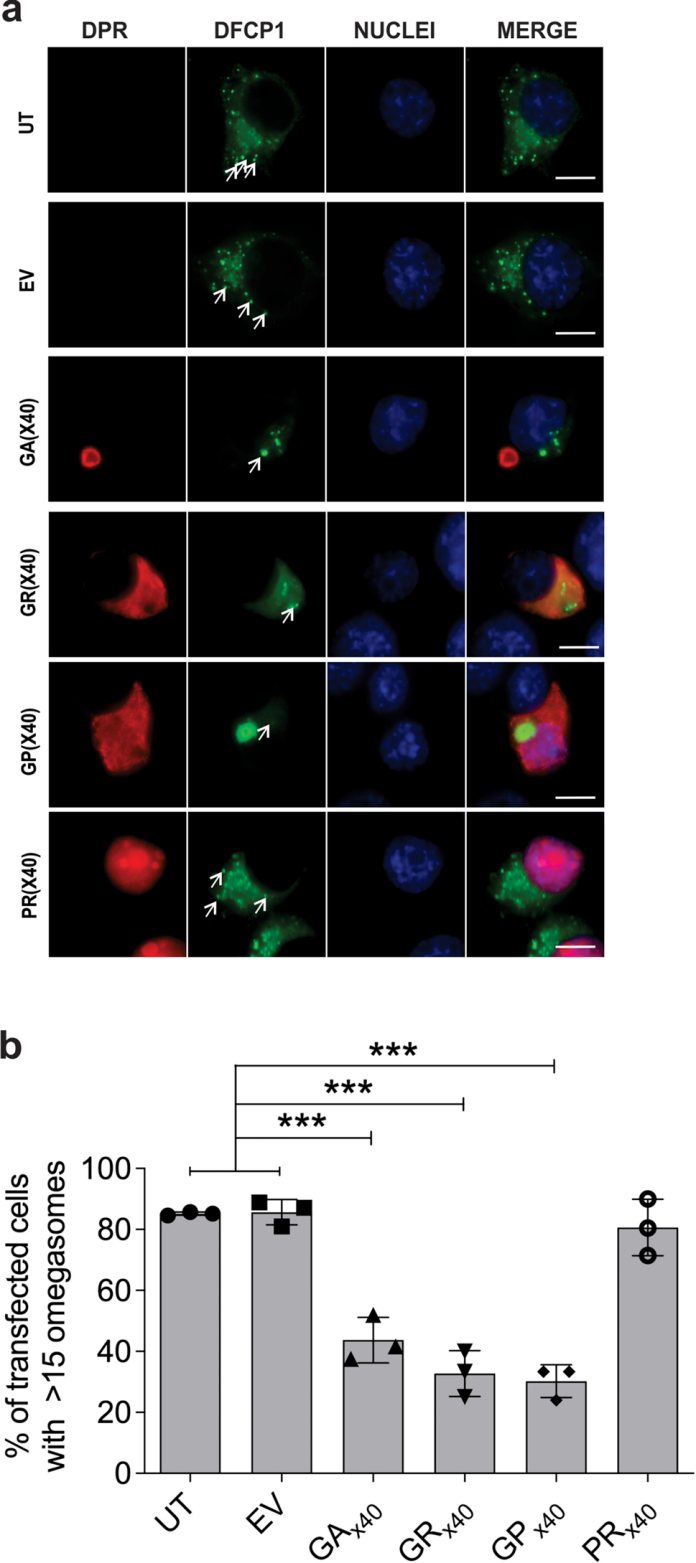
C9orfORF72 DPRs polyGA and polyGP disrupt omegasome formation. (**A**) Fluorescent confocal microscopy of GFP-tagged DFCP1 (double FYVE domain-containing protein) and codon-optimised C9orf72 GA_x40_, GR_x40_, GP_x40_ or PR_x40_ following immunocytochemistry for each DPR. White arrows: omegasomes. Scale bar: 10 µm. (**B**) Quantification of the proportion of transfected cells with > 15 omegasomes in (B). Mean ± SEM, n = 3; one-way ANOVA followed by *post-hoc* Tukey test, ***p < 0.001

Given that the C9orf72 DPRs perturb ER homeostasis, we next examined whether the formation of the omegasome was also inhibited in these cells using a specific marker, double FYVE-containing protein 1 (DFCP1) in pEGFP-C2 [76–79]. Neuro2A cells were co-transfected with constructs encoding C9orf72 DPRs and DFCP1-fused to EGFP. At 48 h post-transfection, immunocytochemistry using anti-DPRs antibodies was performed as above (Fig. 4A). Omegasome formation was quantified by first examining control populations (UT and EV) to determine the average number of omegasomes per cell. Based on this, cells were then scored as positive when > 15 omegasomes per cell were present (identified as GFP-positive punctate structures). A high percentage of cells with 15 + omegasomes were observed in UT (85.2 ± 0.3%) and EV expressing cells (85.6 ± 2.4%). In contrast, significantly (*p* < 0.001) fewer cells with omegasomes were detected in populations expressing C9orf72 DPRs polyGA (43.7 ± 4.3%), polyGR (32.8 ± 4.3%) and polyGP (30.2 ± 3.1%), compared to UT and cells expressing EV (Fig. 4B). However, no significant differences were detected in cells expressing polyPR (80.6 ± 5.4%) compared to UT and EV controls (*p* > 0.05). These data therefore reveal that the C9ORF72 DPRs that inhibit ER-Golgi transport and induce ER stress; polyGA, polyGR and polyGP; disrupt omegasome formation.

### C9orf72 DPRs GA, GR and GP Induce Golgi Fragmentation in Neuro2A Cells

**Fig. 5:**
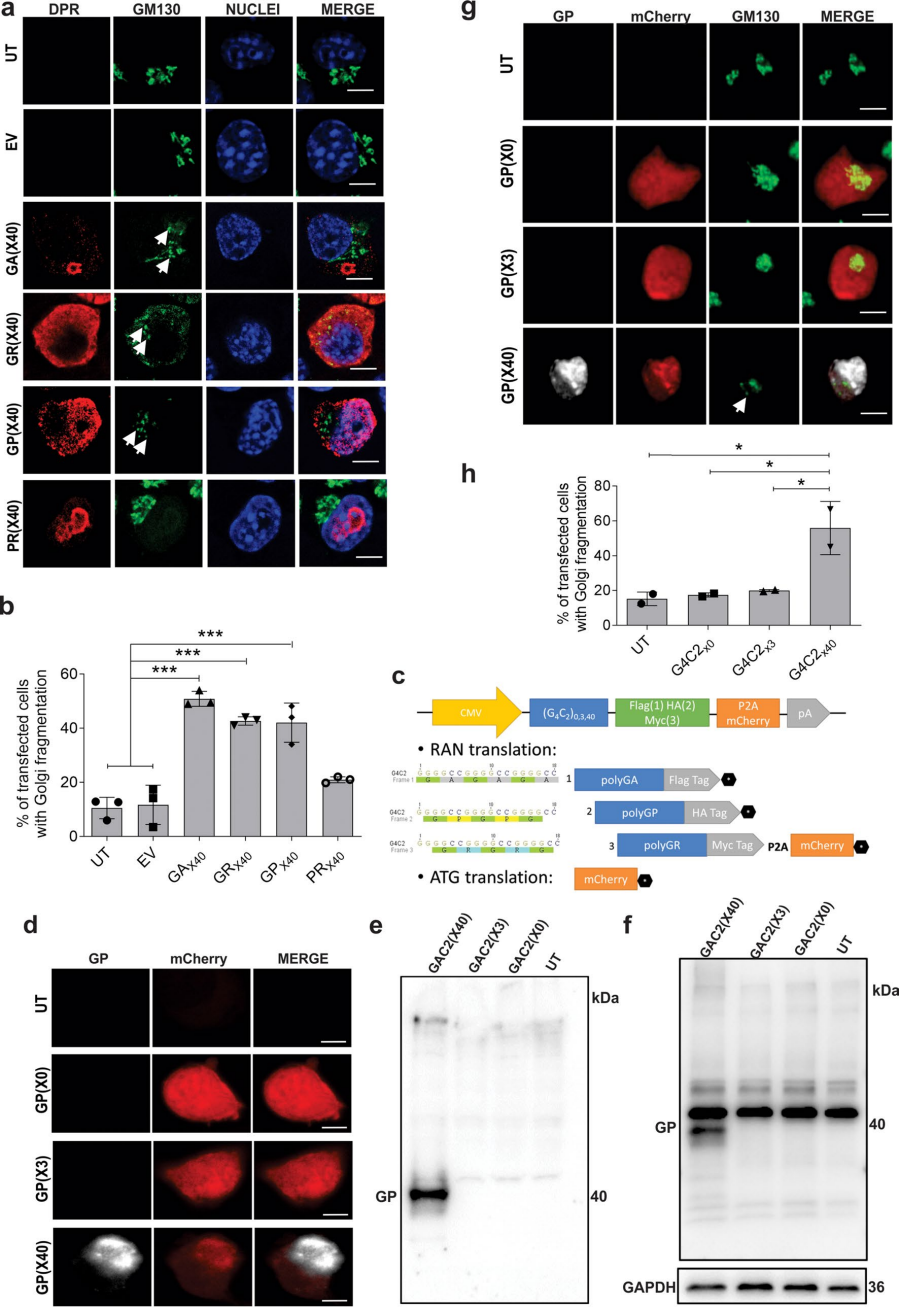
C9orf72 DPRs and RAN expression induce Golgi fragmentation in Neuro2a cells. (**A**) Fluorescent microscopy images of the Golgi apparatus following immunocytochemistry for GM130 in UT cells or populations expressing EV or codon-optimised C9orf72 GA_x40_, GR_x40_, GP_x40_ or PR_x40_ DPRs. Scale bar: 5* µm*. (**B**) Quantification of the proportion of cells expressing pcDNA3.1 empty vector (EV) or C9orf72 DPRs with Golgi fragmentation in (A). Mean ± SEM, n = 3, one-way ANOVA followed by *post-hoc* Tukey test, ***p < 0.001. Significantly more cells with fragmented Golgi were present in populations expressing GA_x40_, GR_x40_, GP_x40_ or PR_x40_ DPRs compared to UT and EV cells. (**C**) Design of constructs for RAN translation of the C9orf72 HRE. Constructs contain either 0, 3 (control) or 40 repeats (G4C2RANx0, G4C2RANx3, G4C2RANx40) with a 3′-3xFLAG, HA and MYC tag (each in a different reading frame) and a mCherry fluorophore, but they lack an ATG start codon. Hence, the C9orf72 HRE can undergo RAN translation in any forward frame. The black hexagon represents a stop codon at the end of the Flag, HA and mCherry tags (if frame 3 is used, translation of the MYC tag is prevented by the mCherry stop codon). P2A sequence induces ribosomal skipping during translation. mCherry sequence has a start codon and thus is also translated in the absence of RAN translation. (**D**) Immunofluorescent microscopy images of cells expressing RAN C9orf72 constructs G4C2_RANx0,_ G4C2_RANx3, or_ G4C2_RANx40_ in Neuro2A cells following immunostaining for polyGP and displaying mCherry. G4C2_RANx40_ produces polyGP by RAN translation in Neuro2A cells. Scale bar: 10* µm*. (**E**) Western blotting of C9orf72 DPRs using anti-polyGP antibody (GP) in insoluble lysate fractions prepared from untransfected (UT) Neuro2A cells or cells expressing mCherry-tagged C9orf72 G4C2_RANx0,_ G4C2_RANx3, or_ G4C2_RANx40_ constructs. PolyGP was expressed in cells transfected with G4C2_RANx40,_ but not G4C2_RANx0_ or G4C2_RANx3_. (**F**) Western blotting of C9orf72 DPRs using an anti-polyGP antibody (GP) in soluble lysate fractions prepared from untransfected (UT) Neuro2A cells or cells expressing mCherry-tagged C9orf72 G4C2_RANx0,_ G4C2_RANx3, or_ G4C2_RANx40_ constructs. GAPDH was used as a loading control. (**G**) Fluorescent confocal microscopy images of mCherry-tagged C9orf72 DPRs and the Golgi apparatus following immunocytochemistry for polyGP and GM130 in untransfected Neuro2A cells (UT), or cells transfected with either G4C2_RANx0,_ G4C2_RANx3, or_ G4C2_RANx40_ constructs. Arrows: fragmented Golgi were identified by condensed punctate structures. Scale bar: 10 µm. (**H**) Quantification of the proportion of cells in (F) with Golgi fragmentation. Mean ± SEM, n = 3; *p < 0.05, one-way ANOVA followed by post-hoc Tukey test. Significantly more cells with fragmented Golgi were present in populations transfected with G4C2_RANx40_, expressing polyGP, compared to those transfected with controls G4C2_RANx0,_ G4C2_RANx3_ or UT cells

Given that defects in both ER-Golgi transport and ER stress induce fragmentation of the Golgi apparatus [28, 80, 81], the morphology of the Golgi was next examined. Neuro2A cells expressing C9orf72 DPRs for 48 h were subjected to immunocytochemistry using anti-DPRs and anti-GM130 antibodies (Fig. 5A). Golgi fragmentation was identified by the formation of condensed, tubulovesicular punctate Golgi structures as previous [36, 82, 83], in contrast to healthy cells, where the Golgi formed a network-like perinuclear structure around the microtubule-organising centre. Quantification revealed few cells displaying Golgi fragmentation in UT (10.5 ± 2.3%) and EV (11.7 ± 4.2%) populations. In contrast, significantly more cells with Golgi fragmentation were detected in populations expressing DPRs polyGA (50.8 ± 1.6%), polyGR (42.7 ± 0.9%) and polyGP (42.0 ± 4.2%), compared to UT and EV cells (Fig. 5B). However, there was no significant difference in cells expressing polyPR compared to controls (20.9 ± 0.6%). These data therefore indicate that C9orf72 DPRs polyGA, polyGR and polyGP induce Golgi fragmentation, consistent with the impairment of ER-Golgi transport and induction of ER stress in these populations.

### C9orf72 Repeat Expansions Undergo DPR RAN Translation in Neuro2A Cells, Producing polyGP and Inducing Golgi Fragmentation

To further validate these results, we next examined RAN translation of the C9orf72 HRE using constructs lacking an ATG start codon. These constructs contained either 0, 3 (both as controls) or 40 repeats (G4C2_RANx0,_ G4C2_RANx3,_ G4C2_RANx40_) with a 3′-3X FLAG, HA and MYC tag (each in a different reading frame) and a mCherry fluorophore. Hence due to the absence of a start codon, the C9orf72 repeats can undergo RAN translation in any forward frame (Fig. 5C). Following transfection of Neuro2A cells for 48 h, mCherry was displayed in all populations, confirming that each construct was expressing correctly (Fig. 5D). Immunocytochemistry using the anti-DPR antibodies revealed that polyGP only, in the absence of the other DPRs, was detected in cells transfected with G4C2_RANx40_ (Fig. 5D). Moreover, this was absent from cells transfected with control constructs expressing G4C2_RANx0_ or G4C2_RANx3_. Similarly, western blotting of the insoluble cell lysate fraction (Fig. 5E) revealed that only cells transfected with the G4C2_RANx40_ construct expressed polyGP, unlike control cells transfected with G4C2_RANx0_ or G4C2_RANx3._ The finding of polyGP in the insoluble fraction is consistent with the presence of misfolded or aggregated protein, as expected. Whilst a non-specific band was detected in soluble lysates that was present in all lanes (Fig. 5F), a specific band for polyGP was detected only in G4C2_RANx40_ populations, representing soluble polyGP (Fig. 5E). In contrast, there was no RAN translation of the repeat into any of the other DPRs except polyGP. These data indicate that expanded G4C2 repeats are RAN translated into polyGP from the G4C2_RANx40_ construct, unlike the control constructs encoding G4C2_RANx0_ or G4C2_RANx3_.

We next examined Neuro2A cells expressing these constructs for the presence of Golgi fragmentation. Immunocytochemistry using anti-GP and anti-GM130 antibodies was performed (Fig. 5G). Quantification of the percentage of transfected cells with Golgi fragmentation revealed few cells in UT (15.3 ± 2.8%) or control populations expressing G4C2_RANx0_ (17.5 ± 0.9%) or G4C2_RANx3_ (20.0 ± 0.4%) (Fig. 5H). In contrast, significantly more cells with Golgi fragmentation were detected in populations expressing G4C2_RANx40_ (55.9 ± 10.8%), compared to UT, G4C2_RANx0_, and G4C2_RANx3_ controls (*p* < 0.05). These data therefore demonstrate that RAN translation, resulting in the production of polyGP, results in fragmentation of the Golgi apparatus. Hence, these findings confirm the results obtained with the DPR constructs.

### Aberrant ER-Derived Vesicles are Present in C9orf72 ALS Fibroblasts and Motor Neurons

Finally, to provide further evidence that ER-Golgi trafficking is perturbed in C9orf72-ALS, we examined ER-derived vesicles in human patient derived fibroblasts and post-mortem patient tissues using immunochemical staining for Sec31A as a marker of ER-derived vesicles and vesicle clusters, in which multiple individual COPII vesicles often cluster together.

**Fig. 6:**
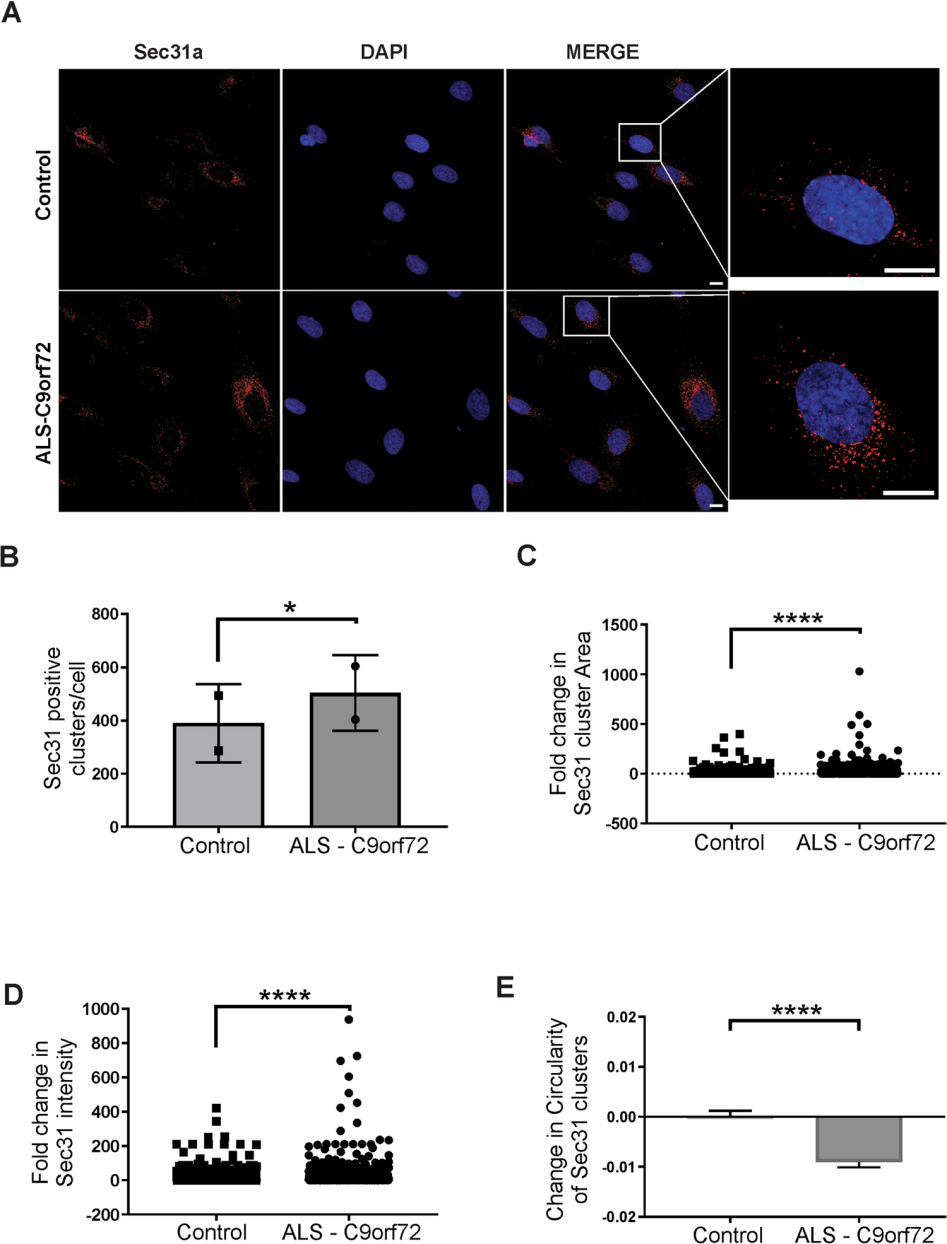
Aberrant Sec31A morphology in ALS-C9orf72 patient fibroblasts. (**A**) Confocal images following immunocytochemistry of Sec31A vesicle clusters in control (n = 2) or ALS-C9orf72 patient (n = 1) derived fibroblasts. (**B**) Quantification of the number of Sec31A-positive clusters, paired t-test, *p < 0.05. (**C**) Quantification of fold-change in area of each Sec31A positive vesicular cluster in C9orf72 fibroblasts relative to average vesicle area of control fibroblasts. ****p < 0.0001. (**D**) Quantification of fold-change in intensity of Sec31A staining in each cluster in C9orf72 fibroblasts relative to average staining intensity of control fibroblasts. ****p < 0.0001. (**E**) Quantification of change in circularity of detected clusters. Circularity was obtained from ImageJ (ranging from 0, – not circular, to 1, – fully circular) and the difference to average circularity of control fibroblasts was calculated. ****p < 0.0001. Mean ± SEM of individual clusters depicted. (**B**)-(**E**) data points represent technical replicates using two different antibodies to Sec31A

In C9orf72 fibroblasts, the Sec31A-positive vesicular clusters were more numerous compared to those from control fibroblasts (*p* = 0.0192; Fig. 6B). The clusters were also larger with enhanced staining (*p* < 0.0001; Fig. 6C-D). The morphology of the clusters was also altered in that they were less circular compared to control fibroblasts (*p* < 0.0001; Fig. 6E). Thus, in the C9orf72 patient fibroblasts the vesicle clusters were abnormal, consistent with dysregulation of ER-Golgi transport by the C9orf72 repeat expansion.

**Fig. 7:**
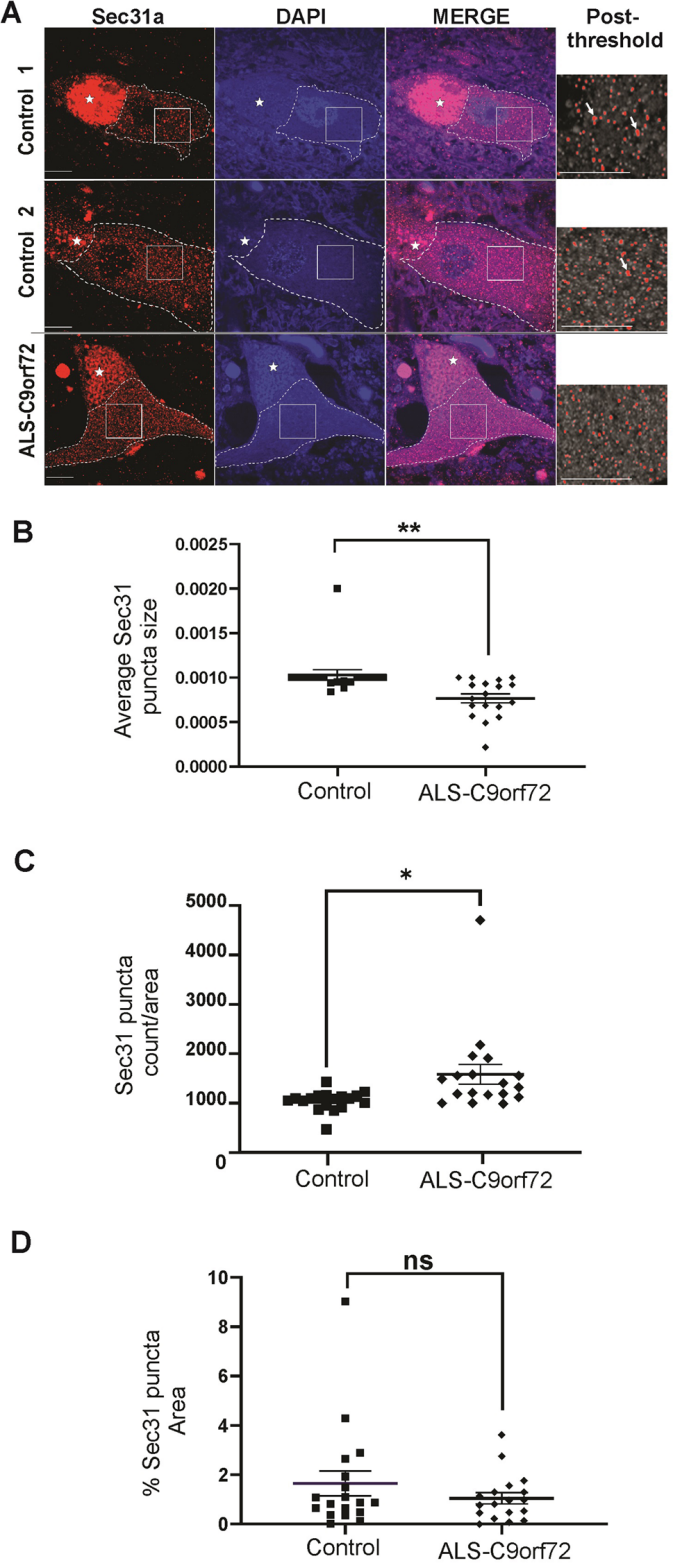
Aberrant Sec31A morphology in ALS-C9orf72 patient spinal cord tissues. (**A**) Confocal images following immunohistochemistry for Sec31A of control or ALS-C9orf72 patient spinal cords (n = 3 of each). Last panel shows staining after threshold setup in Fiji Image J. Scale bar: 20 µm. Star: lipofuscin area was excluded from the region analysed. Dashed area: region of interest for Sec31A analysis. Neurons were identified by their characteristic size and morphology. Arrows: larger Sec31A puncta were observed in control patients compared to ALS-C9orf72 patient motor neurons. (**B**) Quantification of the average Sec31A puncta size. At least six neurons from each control and ALS-C9orf72 patient were analysed. Mean ± SEM from three control and three patient tissues; student’s *t* test, ** *p* < 0.01. Sec31A puncta were significantly smaller in ALS-C9orf72 patient neurons compared to control cells. (**C**) Quantification of Sec31A puncta count/area. Mean ± SEM from three control and three patients; student’s *t* test, * *p* < 0.05. ALS-C9orf72 neurons displayed significantly more Sec31A puncta per area compared to control patient cells. (**D**) Quantification of the percentage area of total Sec31A puncta between controls and patient motor neurons, ns = non significant

In spinal cord sections from three ALS-C9orf72 patients and three neurologically normal controls, immunohistochemistry was performed. Motor neurons were identified by their characteristic size and morphology. In both ALS and control motor neurons, Sec31A displayed its typical punctate staining, as expected. However, in C9orf72 motor neurons, Sec31A-positive vesicular clusters were more numerous and smaller compared to control cells (Fig. 7A). Using the Fiji Image J particle analysis function, we examined Sec 31 particle size and Sec31A-puncta area in C9orf72 and control motor neurons. We performed two statistical analyses methods, a student’s* t*-test and a linear mixed effect model analysis, which takes hierarchical data structure into consideration (neurons within individual cases), unlike the *t*-test. Both particle size and the number of Sec31A-puncta per area were significantly different in C9orf72 cells compared to control cells using a student’s* t*-test (*p* = 0.0018 and *p* = 0.0135, respectively, Fig. 7B, C). However, these data were not statistically significant when analysed using the more stringent linear mixed effect model (particle size *p* = 0.07098; puncta per area *p* = 0.1589). A possible explanation for these differences is that the most severely affected motor neurons would have already degenerated in post-mortem tissues, and the remaining neurons may not display the pathological consequences as strongly. Hence, a trend towards decreased particle size, and more Sec31A-puncta per area in C9orf72 motor neurons compared to those of control patients, was detected.

Interestingly, we did not observe any significant changes in percentage area of total Sec31A puncta between control and patient using either statistical method (student’s* t*-test *p* = 0.2827; linear mixed effect model *p* = 0.3763), suggesting that despite the difference in puncta size and number, the area of Sec31A puncta were similar between the control and ALS patients (Fig. 7D). To confirm that post-mortem fixation delay did not affect immunoreactivity and the localisation of Sec31A, we examined whether there were any significant differences in Sec 31 puncta count/area, % area and average size between the two patients with post-mortem intervals (PMIs) at the top and bottom of range in this study: ALS-C9orf72 1 and ALS-C9orf72 3 (PMI 7 h and 99 h, respectively). We demonstrated that there were no significant differences between ALS-C9orf72 1 and ALS-C9orf72 3 in any of the parameters tested (Sec 31 puncta count/area (p = 0.6542), % area (p = 0.1587) and average size (p = 0.0903). Together, this finding demonstrated a trend toward perturbation of Sec31A-positive ER-derived puncta in C9orf72 motor neurons, supporting the impairment of ER-Golgi transport by the C9orf72 HRE.

## Discussion

The molecular mechanisms by which *C9orf72* HREs induce neurodegeneration remain unclear, although C9orf72 loss-of-function through haploinsufficiency, or toxic gain-of-function by either repeat RNA or DPRs, are implicated [84]. In this study, we describe novel molecular gain-of-function mechanisms induced by the C9orf72 DPRs – impairment of ER–Golgi transport and omegasome formation, Golgi fragmentation, and  induction of ER stress. We also confirm that Golgi fragmentation is present in cells undergoing RAN translation of the C9orf72 HRE. These data therefore provide new insights into pathophysiology involving the ER and Golgi compartments.

The five unique DPRs generated from the bidirectionally transcribed C9orf72 HRE were found to have different cellular localisations, consistent with previous studies. Here we detected polyGA predominately in the cytoplasm. Similarly, polyGA formed inclusions mainly in the cytoplasm in mouse primary neurons and in the cortex [64] and spinal cord tissues [65] of C9orf72-ALS patients. In contrast, and similar to the findings obtained here, polyPR DPRs were almost exclusively localised within the nucleus and adjacent to the nucleolus in either HeLa cells or rat primary hippocampal neurons expressing polyPR, and in the frontal cortex and spinal cord of patients with *C9orf72* mutations [64–66]. Previously, polyGR and polyGP were expressed both in the nucleus and within the cytoplasm [22, 65, 66]. Here, we detected polyGP predominately in the cytoplasm whereas polyGR was present almost exclusively in the cytoplasm. Hence each DPR_x40_ has unique expression and cellular localisation in neuroblastoma cell lines, consistent with previous studies [64].

The normal cellular function of C9orf72 is thought to involve the regulation of membrane transport events, particularly endosomal trafficking [42, 54, 85]. We and others previously demonstrated that C9orf72 associates with multiple members of the Rab small GTPase family that mediate vesicular trafficking, including Rab1A [42, 43], Rab7 [86], and Rab11 [42]. C9orf72 is also involved in the control of presynaptic vesicle trafficking [87]. Compromised extracellular vesicle secretion and multivesicular endosome formation has been detected in fibroblasts and iPSC-derived motor neurons [86]. C9orf72 DPRs are also known to perturb nucleocytoplasmic trafficking [88–91], another form of cellular transport. However, impairment of trafficking in the early secretory pathway, between the ER and Golgi compartments, has not been previously described in C9orf72-ALS.

ER–Golgi transport is a vital gateway to all proteins entering the secretory pathway. Correctly folded proteins exit the ER in COPII-coated vesicles, which then fuse with the Golgi before they are targeted to their specific destinations [92, 93]. ER–Golgi transport is therefore crucial in maintaining protein secretion and thus cellular function. Here, we demonstrate that the C9orf72 DPRs perturb ER-Golgi transport in neuronal cells. We detected this using a classical marker of secretory protein transport, the temperature-sensitive mutant VSVG^ts045^, because more was retained in the ER and less was transported to the Golgi in C9orf72 DPRs-expressing cells compared to controls. We also demonstrated inhibition of ER-Golgi trafficking in C9orf72 patient fibroblasts, confirming that this is not due to non-specific protein over-expression. Furthermore, these fibroblasts expressed significantly less C9orf72 protein, implying that haploinsufficiency may also combine with inhibition of transport induced by the toxic DPRs in ALS. Moreover, we detected perturbed ER-derived vesicles in spinal cord motor neurons and fibroblasts from C9orf72 ALS patients compared to controls, confirming that the C9orf72 HRE perturbs ER-Golgi vesicular trafficking. Although this change was significant only with student’s *t*-test and not the more stringent linear mixed effect model, we detected a trend towards smaller vesicles in spinal cord neurons of patients compared to controls, suggesting that C9orf72 HRE disturbs COPII vesicle formation, leading to defects in transport capacity. However, it is possible that motor neurons displaying the strongest phenotype will have already degenerated in human post-mortem tissues. In post-mortem samples it is not possible to detect early cellular pathogenic events, and thus to characterise the properties of Sec31A vesicles in the initial stages of disease pathogenesis in motor neurons. However, as in spinal cords, significant differences in Sec31A vesicle clusters were detected between fibroblasts from C9orf72-ALS and control patients. Significantly more Sec31A vesicle clusters were detected in C9orf72-fibroblasts, and these vesicles were abnormal, although they were larger in size, rather than smaller, as in post-mortem tissues. These differences may reflect the significant functional differences in the two cell types being examined and that the former cells are not affected pathologically in ALS. Fibroblasts are specialised secretory cells producing large quantities of protein components of the extracellular matrix, including collagen, elastin and glycosaminoglycans. In most cell types, COPII-vesicles are typically ~ 60-80 nm in diameter, but to secrete procollagen, COPII vesicles need to increase their size to 300-400 nm in cells producing collagen. Moreover, Sec31A mono-ubiquitination drives the formation of these large COPII-vesicles to accommodate the unusually bulky collagen cargo  [94]. Thus, in both fibroblasts and spinal motor neurons, we detected more Sec31A vesicles and found evidence that they are abnormal in C9orf72-ALS. In cells that secrete significant quantities of large cargo proteins (fibroblasts) Sec31A vesicle clusters are larger than normal, but they are smaller than controls in the remaining spinal motor neurons of patient tissues at disease end-stage. An alternative explanation for the different Sec31A staining patterns between fibroblasts and spinal cord motor neurons is the reported diversity of the latter, and existence of multiple subtypes [95, 96]. Hence it is possible that specific subtypes of spinal cord motor neurons may be more vulnerable to C9orf72-DPRs than others.

It will be worthwhile in the future to examine Sec31A early in disease course in mouse models, where its expression can be examined temporally during disease progression. However, currently the existing C9orf72-ALS mouse models do not fully recapitulate all features of ALS [97], hence this was not examined here. We previously established that GFP only and other non-ALS-associated mutant proteins do not perturb ER-Golgi transport, demonstrating that this process is not due to protein over-expression systems [81]. Other ALS-associated mutants – SOD1, TDP-43, UBQLN2 and FUS – also specifically inhibit ER–Golgi transport in neuronal cells, providing evidence that this is a common pathophysiological mechanism amongst diverse forms of ALS and may provide an avenue for therapeutic development [28, 81].

This study also shows that polyGA, polyGR and polyGP DPRs induce Golgi fragmentation [82, 83]. We also confirmed that this feature is induced by RAN translation because expanded G4C2 repeats were translated into polyGP, and Golgi fragmentation was detected in these cells. Fragmentation of the neuronal Golgi apparatus is a well described pathological characteristic associated with ALS [28, 83, 98–100] that has been detected in sporadic ALS patient motor neurons [100], in animal disease models based on mutant SOD1 [101, 102] or expressing mutant TDP-43^M337V^ [103], and in cells expressing mutant forms of SOD1 or FUS [104]. The structure of the Golgi stacks is regulated by balanced anterograde and retrograde transport through this organelle [76] and impairment of ER-to-Golgi transport results in Golgi fragmentation [74, 75]. Hence, our results are the first to link inhibition of ER–Golgi transport by C9orf72 DPRs to Golgi fragmentation, confirming that disruption to the early part of the secretory pathway is present in C9orf72-mediated ALS.

We also detected activation of the UPR in cells expressing C9orf72 DPRs, detected by spliced XBP-1 and the presence of pro-apoptotic factor CHOP in the nucleus. Activation of ER stress is a widely described feature in ALS [70, 105–107], which first starts in the most vulnerable neurons in the SOD1-G93A mouse model before disease onset [106]. Consistent with our observations, it was previously shown that C9orf72 DPRs induce ER stress, leading to apoptosis [108–111]. Expression of polyGA in primary neurons was accompanied by induction of ER stress and caspase-3 activation [112], and polyPR can elicit an ER-stress response in SH-SY5Y cells and in neuron cultures expressing polyPR [113, 114]. Furthermore, ER stress inhibitors salubrinal and TUDCA provide protection against polyGA-induced toxicity [112]. Increased ER calcium and vulnerability to tunicamycin-induced ER stress has also been observed in C9orf72 iPSC-derived motor neurons   [108, 115].  In contrast iPSC-derived neurons positive for a telencephalon marker [116] were not sensitive to tunicamycin, implying that C9orf72-induced ER stress is more pronounced in motor neurons compared to other types of neurons. Induction of ER stress by polyGA was linked to impairment of GRP75 at the MAM compartment [111]. Additionally, ER stress can increase RAN translation [117, 118], suggesting the existence of a possible feed-forward loop. Increased mRNA levels of activating transcription factor 4 (ATF4) and CHOP were also detected in the frontal cortex of C9orf72-ALS patients, indicating that ER stress also occurs in human patients [112]. Together these data imply that ER stress is an active mechanism causing neuronal death in C9orf72-associated ALS/FTD. However, the mechanism responsible for inducing ER stress in these forms of ALS/FTD still remain unclear.

Perturbations in ER-Golgi transport by the C9orf72 DPRs would lead to the accumulation of misfolded secretory proteins in the ER, providing a possible explanation for the induction of ER stress we detected here [34, 119–121]. In previous studies, we showed that ER stress occurs subsequent to, or coincident with, inhibition of ER-Golgi trafficking in cells expressing ALS-associated mutant forms of SOD1, TDP-43 or FUS [81]. Interestingly impairment of ER-Golgi transport, induction of ER stress and Golgi fragmentation, were all detected in cells expressing the DPRs with the most cytoplasmic localisation—polyGA, polyGR or polyGP—but were absent in cells expressing polyPR, which was localised mainly in the nucleus in this study. Hence these results imply that impairment of trafficking is mediated by DPRs with significant expression in the cytoplasm, rather than nucleus. Our observations are therefore consistent with previous studies implying that inhibition of ER-Golgi trafficking by mutant SOD1, TDP-43 and FUS induces ER stress from the cytoplasm [81]. However, we cannot rule out the possibility that ER stress might be directly triggered directly by the C9orf72 DPRs within the ER. Thus, further analysis in this area is required.

We also found that formation of the omegasome, detected by quantification of DFCP1-specific punctate structures, is also impaired by the C9orf72 DPRs. Previous studies have linked dysfunction of the trans-Golgi network, inhibition of autophagy and protein degradation pathways [122]. Several lines of evidence indicate that C9orf72 regulates autophagy [43, 123–125] and autophagy is disrupted in C9orf72-deficient neurons and in iPSCs derived-MNs from C9orf72-ALS patients [123, 126, 127]. Our findings therefore imply that autophagy impairment by C9orf72 DPRs thus originates from the ER, by inhibition of omegasome formation.

In summary, this study identifies novel molecular mechanisms induced by the C9orf72 HRE involving the ER and Golgi compartments. Whilst the exact sequence of events remains to be determined, disruption of ER to Golgi transport by C9orf72 DPRs links together ER stress, omegasome formation, and Golgi fragmentation and it provides a possible mechanism linking together alterations of the folding capacity of the ER and haploinsufficiency. This study also provides further evidence that dysfunction to the early secretory pathway is a common upstream pathological event in diverse forms of ALS/FTD.

## Supplementary Information

Below is the link to the electronic supplementary material.

Supplementary file1 (DOCX 177 KB)

## Data Availability

The datasets generated during and analysed during the current study are available from the corresponding author on reasonable request.
