# Supplementary material for: C9orf72-Associated Dipeptide Repeat Expansions Perturb ER-Golgi Vesicular Trafficking, Inducing Golgi Fragmentation and ER Stress, in ALS/FTD
Source: Mol Neurobiol. 2024 May 9;61(12):10318–38. doi: 10.1007/s12035-024-04187-4 (PMC11584443; doi:10.1007/s12035-024-04187-4)

**Supplementary Information**

**Molecular Neurobiology**

**The C9orf72 repeat expansion inhibits ER-Golgi vesicular trafficking, inducing Golgi fragmentation and ER stress, in ALS/FTD.**

Jessica Sultana^1^, Audrey M.G. Ragagnin^1^, Sonam Parakh^1^, Sayanthooran Saravanabavan^1^, Kai Ying Soo^1^, Marta Vidal^1^, Cyril Jones Jagaraj, Kunjie Ding^1^, Sharlynn Wu^1^, Sina Shadar^1^, Emily K Don^1^, Anand Deva^2^, Garth Nicholson^1, 3^, Dominic B. Rowe^1^, Ian Blair^1^, Shu Yang^1^, Julie D Atkin^1, 4, *^

^1^Motor Neuron Disease Research Centre, Faculty of Medicine and Health Sciences, Macquarie University, Sydney, Australia.

^2^Department of Plastic and Reconstructive Surgery, Macquarie University, and The Integrated Specialist Healthcare Education and Research Foundation, Sydney, Australia.

^3^ ANZAC Research Institute, Concord Hospital, University of Sydney, Sydney, NSW, Australia.

^4^La Trobe Institute for Molecular Science, La Trobe University, Bundoora, Melbourne, Australia.

*Correspondence and request for materials should be addressed to J.D.A (email: [julie.atkin@mq.edu.au](mailto:julie.atkin@mq.edu.au)). ORCID: 0000-0003-2427-499X

**Supplementary Information**

(**S1**) Fluorescent microscopy images of codon-optimised FLAG-tagged C9orf72 polyGA, polyGR, polyGP or polyPR DPRs_x40_ following immunocytochemistry for C9orf72 DPRs and Hoechst staining. Cells were identified as apoptotic when the nuclei were condensed. Arrows: condensed nucleus. Scale bar: 10 *µ*m.

(**S2**) Quantification of the proportion of transfected cells in (**S1**) with nuclear condensation, indicating apoptosis is underway. Mean ± SEM, n=3; one-way ANOVA followed by *post-hoc* Tukey test, **p*<0.05, ****p*<0.001 vs UT or EV, #*p*<0.05, ##*p*<0.01 vs DPRs.


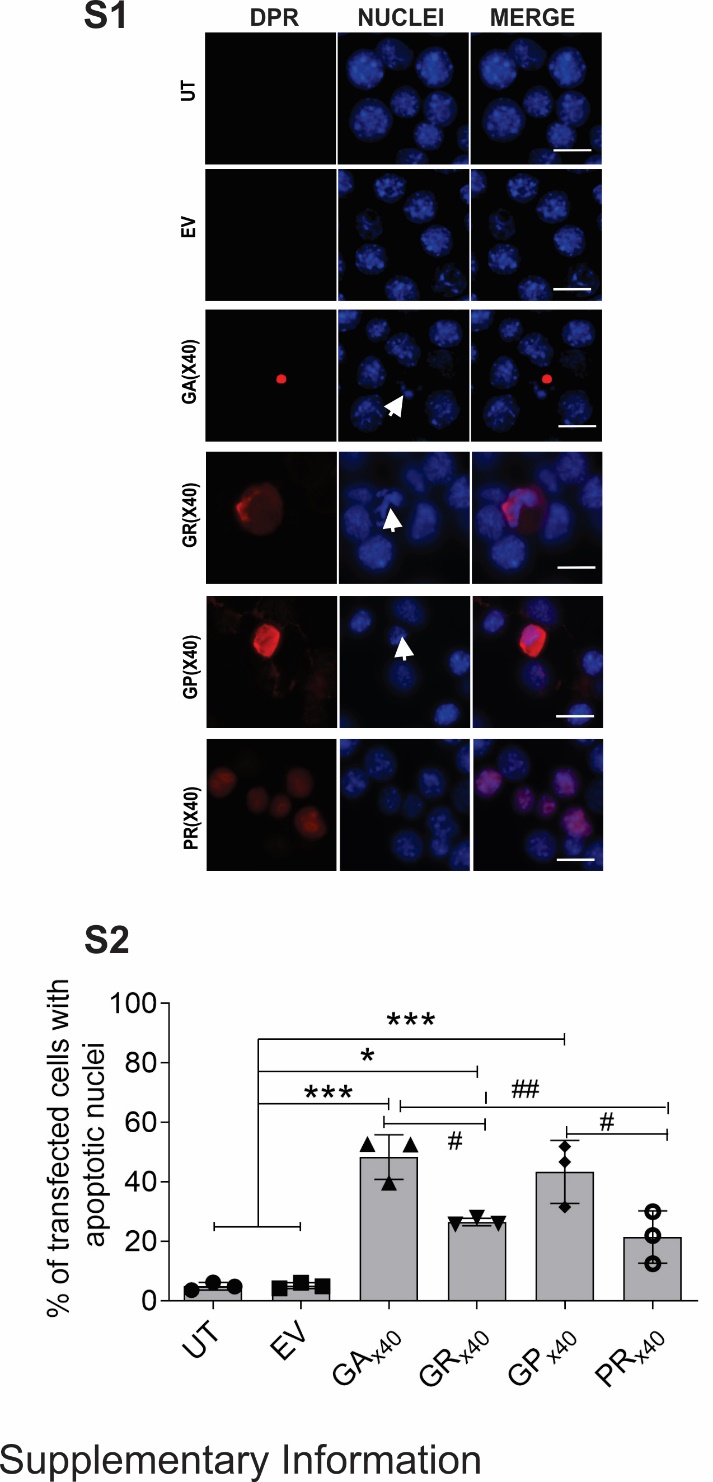

Supplement: Supplementary file 1 — Supplementary file1 (DOCX 177 KB) [file 12035_2024_4187_MOESM1_ESM.docx]
